# Patterns of antimicrobial agent prescription in a sentinel population of canine and feline veterinary practices in the United Kingdom

**DOI:** 10.1016/j.tvjl.2017.03.010

**Published:** 2017-06

**Authors:** D.A. Singleton, F. Sánchez-Vizcaíno, S. Dawson, P.H. Jones, P.J.M. Noble, G.L. Pinchbeck, N.J. Williams, A.D. Radford

**Affiliations:** a Institute of Infection and Global Health, University of Liverpool, Leahurst Campus, Chester High Road, Neston, CH64 7TE, United Kingdom; b National Institute for Health Research, Health Protection Research Unit in Emerging and Zoonotic Infections, The Farr Institute @ HeRC, University of Liverpool, Waterhouse Building, Liverpool, L69 3GL, United Kingdom; c Institute of Veterinary Science, University of Liverpool, Leahurst Campus, Chester High Road, Neston, CH64 7TE, United Kingdom

**Keywords:** Canine, Feline, Antimicrobial resistance, Antibiotic prescribing practices, Surveillance

## Abstract

Antimicrobial resistance is an increasingly important global health threat and the use of antimicrobial agents is a key risk factor in its development. This study describes antimicrobial agent prescription (AAP) patterns over a 2 year period using electronic health records (EHRs) from booked consultations in a network of 457 sentinel veterinary premises in the United Kingdom. A semi-automated classification methodology was used to map practitioner defined product codes in 918,333 EHRs from 413,870 dogs and 352,730 EHRs from 200,541 cats, including 289,789 AAPs. AAP as a proportion of total booked consultations was more frequent in dogs (18.8%, 95% confidence interval, CI, 18.2–19.4) than cats (17.5%, 95% CI 16.9–18.1). Prescription of topical antimicrobial agents was more frequent in dogs (7.4%, 95% CI 7.2–7.7) than cats (3.2%, 95% CI 3.1–3.3), whilst prescription of systemic antimicrobial agents was more frequent in cats (14.8%, 95% CI 14.2–15.4) than dogs (12.2%, 95% CI 11.7–12.7). A decreasing temporal pattern was identified for prescription of systemic antimicrobial agents in dogs and cats. Premises which prescribed antimicrobial agents frequently for dogs also prescribed frequently for cats. AAP was most frequent during pruritus consultations in dogs and trauma consultations in cats. Clavulanic acid potentiated amoxicillin was the most frequently prescribed antimicrobial agent in dogs (28.6% of prescriptions, 95% CI 27.4–29.8), whereas cefovecin, a third generation cephalosporin, was the most frequently prescribed antimicrobial agent in cats (36.2%, 95% CI 33.9–38.5). This study demonstrated patterns in AAP over time and for different conditions in a population of companion animals in the United Kingdom.

## Highlights

• Antimicrobial agent prescription was monitored in a large UK population of cats and dogs over a 2 year period (2014–2016).
• Systemic antimicrobial agents were prescribed more frequently to cats; topical prescription was more frequent in dogs.
• A temporal reduction (2014–2016) in antimicrobial agent prescription was observed in both cats and dogs in this population.
• Premises which prescribed antimicrobial agents commonly to cats generally also prescribed commonly to dogs.
• The most frequently prescribed antibiotics were cefovecin in cats and clavulanic acid potentiated amoxicillin in dogs.

## Introduction

Antimicrobial resistance (AMR) is widely recognised as an increasingly important global health threat.^1^^,^^2^^,^^3^^,^^4^ Evidence of transmission of bacterial resistance amongst human beings, livestock (Cuny et al., 2015) and companion animals^1^ (Zhang et al., 2016) demonstrates the necessity of a ‘one health’ approach to preserve treatment efficacy.^2^ Although use of antimicrobial agents selects for and promotes transfer of resistance (Rantala et al., 2004, Magalhaes et al., 2010, Cantón and Bryan, 2012), data on antimicrobial agent prescription (AAP) to date are limited in animals.

Antimicrobial agents are frequently prescribed in dogs and cats (Mateus et al., 2011, Radford et al., 2011, Buckland et al., 2016), and there is evidence of development of resistance in response to treatment^1^ (Trott et al., 2004), and transmission of antimicrobial resistant isolates between human beings and pets (Johnson et al., 2008a, Johnson et al., 2008b, Zhang et al., 2016). Specific guidance for practice level prescription policies have been published^5^^,^^6^ (Beco et al., 2013a, Beco et al., 2013b); however, there is a need to understand how these are being applied in practice.

Data on human AAP in the United Kingdom (UK) are freely available, in part because of a national health system.^7^ For animals, the Veterinary Medicines Directorate (VMD) is constructing a central body collating data on AAP for the UK; however data currently available cannot identify antimicrobial agents administered under the cascade prescribing system, which species they have been prescribed to, practice level prescription variability or why the antimicrobial agents were prescribed.^8^ Advances in veterinary health informatics provides opportunities to fill this gap, particularly for companion animals where Electronic Health Records (EHR) are most developed and accessible (O’Neill et al., 2014a).

Early studies of companion animal AAP in the UK were limited in size, but have consistently pointed to frequent use of β-lactams (Mateus et al., 2011, Radford et al., 2011). More recently, using a much larger data set, 25% of dogs and 21% of cats seen at veterinary practices received at least one AAP over a 2 year period (2012–2014), the most frequent being penicillins and cephalosporins (Buckland et al., 2016). Whilst such ‘big data’ studies have started to report on AAP, this study aims to describe a near real-time, on-going, AAP surveillance system from a diverse range of veterinary premises (*n* = 457) that also consider AAP in a broad range of practitioner defined clinical presentations.

## Materials and methods

### Data collection

The Small Animal Veterinary Surveillance Network (SAVSNET) collected EHRs in near real-time from booked consultations in volunteer UK veterinary practices (1 April 2014–31 March 2016). A full description of the data collection protocol has been described by Sánchez-Vizcaíno et al. (2015). A practice (*n* = 216) was defined as a single business, whereas premise(s) (*n* = 457) included all branches that form a practice (see Appendix: Supplementary Fig. 1). Before submitting each consultation to SAVSNET, the practitioner selected one of 10 main presenting complaints (MPCs), consisting of a pre-determined list grouped into healthy, unhealthy and post-operative categories (see Appendix: Supplementary Table 1). The EHR further included product codes as text strings defined by individual practices.

### Antimicrobial agent identification

The product codes of the EHR were utilised to identify AAP. A set of 52,267 codes (extracted 26 August 2015) were manually categorised. Pharmaceutical products were defined with reference to the VMD’s Product Information Database for veterinary authorised products, and the electronic Medicines Compendium (Datapharm Communications) for human authorised products. An identifying string was ascribed to each antimicrobial agent product and was used to identify the product code. This process was reiterated until all pharmaceutical and non-pharmaceutical product codes were classified to further validate antimicrobial agent identification. When applied to the complete list of 95,709 codes (extracted 31 March 2016), 416 antimicrobial agent identifying strings were utilised.

Where possible, product codes for antimicrobial agents were further characterised to specific species authorisation and administration by systemic (oral or injectable) or topical (topical, aural or ocular) routes. Whilst not all products were authorised for human use at the time of the study, we considered all fluoroquinolones, macrolides and third generation cephalosporins as highest priority critically important antimicrobial agents (HPCIA), as defined by the World Health Organization (WHO).^9^

### Statistical analysis

Consultation and prescription-level proportions and confidence intervals were calculated to adjust for clustering (bootstrap method, *n* = 5000 samples) within premises and at animal level within practices.^10^ Pearson correlations (*t*-test to reject null hypothesis) were performed to explore prescription frequency for dog and cat total, systemic and topical AAP as a proportion of total submitted consultations for each premises. Paired *t*-tests with Bonferroni corrections were used for a matched pairs premises level sample to investigate total, systemic and topical AAP as a proportion of total submitted consultations for each MPC.

A mixed effects binomial regression model, incorporating practice and premise as random effects, was utilised to examine quarterly variation in total, systemic and topical canine and feline AAP as a proportion of total consultations. The variable time was categorised as an ordinal variable into quarters of the year (Q1, Q2, Q3 and Q4) and included as a fixed effect. Quarter was codified using two contrasting coding systems: (1) an orthogonal polynomial method^11^ to analyse for overall trend (see Appendix: Supplementary Table 2); and (2) a backward differencing method^12^ to investigate quarter-by-quarter variation in a backward pairwise manner (e.g. Q1 2016 compared with Q4 2015). A further model was fitted for canine and feline HPCIA prescription as a proportion of total AAP. A likelihood ratio test (LRT) indicated that including practice and premise as random effects in all models provided the best fit. Statistical significance was defined as *P* < 0.05 and all analyses were carried out using R (version 3.2.3).^13^

## Results

A total of 918,333 canine EHRs (from 413,870 dogs) and 352,730 feline EHRs (from 200,541 cats) were obtained from 216 veterinary practices (457 premises) from 1 April 2014 to 31 March 2016.

### Consultation and animal level

**Fig. 1:**
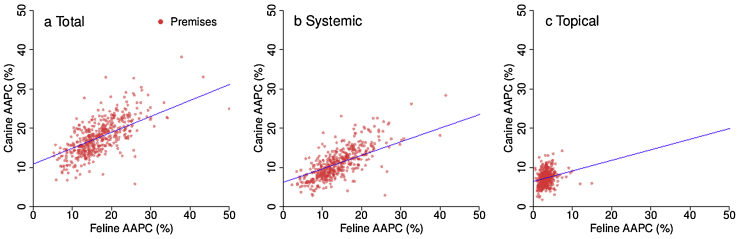
Comparison of canine and feline antimicrobial agent prescription as a percentage of total consultations (AAPC) by premises (*n* = 457) split by (a) total, (b) systemic and (c) topical antimicrobial agent prescription.

The percentage of consultations where at least one antimicrobial agent was prescribed (AAPC) was significantly greater for dogs (18.8%, 95% confidence interval, CI, 18.2–19.4) than cats (17.5%, 95% CI 16.9–18.1). Systemic AAPC was significantly less frequent in dogs (12.2%, 95% CI 11.7–12.7) than cats (14.8%, 95% CI 14.2–15.4), representing 64.9% (95% CI 63.8–66.0) and 84.5% (95% CI 83.9–85.2) of total canine and feline AAPC, respectively (paired *t*-test; *P <* 0.001). Topical AAPC was significantly more frequent in dogs (7.4% of consultations, 95% CI 7.2–7.7) than cats (3.2%, 95% CI 3.1–3.3), representing 39.6% (95% CI 38.5–40.6) and 18.3% (95% CI 17.7–19.0) of AAPC, respectively (*P <* 0.001). Dogs and cats were co-prescribed systemic and topical antimicrobial agents in 0.87% (95% CI 0.84–0.94) and 0.59% (95% CI 0.54–0.64) of total consultations, respectively. Significant positive correlations were found between dogs and cats at premise level for total (0.62, 95% CI 0.56–0.67, *P <* 0.001), systemic (0.61, 95% CI 0.54–0.66, *P <* 0.001) and topical (0.21, 95% CI 0.12–0.30, *P <* 0.001) AAPC (Fig. 1).

**Fig. 2:**
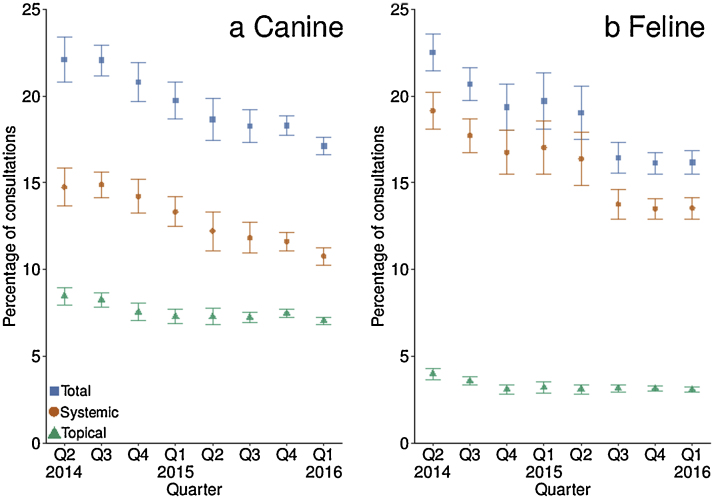
Comparison of (a) canine (*n* = 918,333 electronic health records) and (b) feline (*n* = 352,730) total, systemic and topical antimicrobial agent prescription as a percentage of total consultations (95% confidence interval) by quarter (Q2 2014–Q1 2016).

Fig. 2 shows AAPC categorised by quarter. A significant negative linear trend was observed for canine total and systemic AAPC, and feline total, systemic and topical AAPC (*P <* 0.001; see Appendix: Supplementary Table 3). A significant negative trend by quarter was observed for canine topical AAPC (*P <* 0.001). Results of quarter-by-quarter comparison models can be found in Supplementary Table 4 (see Appendix).

Over the 2 year period, at the animal level, 28.4% (95% CI 27.2–29.7) of dogs were prescribed an antimicrobial agent, compared with 23.3% (95% CI 22.3–24.4) of cats. When route of administration was considered, 19.6% (95% CI 18.4–20.7) of dogs and 20.0% (18.9–21.0) of cats were prescribed a systemic antimicrobial agent, and 12.9% (95% CI 12.3–13.5) of dogs and 5.0% (95% CI 4.7–5.2) of cats were prescribed a topical antimicrobial agent.

Total AAPC was 35.5% (95% CI 34.5–36.5) of unhealthy dogs, 35.1% (95% CI 34.1–36.1) of unhealthy cats, 7.4% (95% CI 6.7–8.0) of healthy dogs and 5.5% (95% CI 4.9–6.2) of healthy cats. Systemic AAPC was more frequent in unhealthy cats (30.5%, 95% CI 29.5–31.5) than unhealthy dogs (24.1%, 95% CI 23.1–25.0). The MPCs with the highest frequencies of AAPC were pruritus in dogs (51.0%, 95% CI 49.8–52.2) and trauma in cats (53.5%, 95% CI 52.1–54.8). Antimicrobial agents were prescribed in a significantly greater proportion of dogs than cats for gastroenteric (*P <* 0.001), pruritus (*P <* 0.001), kidney disease (*P <* 0.001), other unwell (*P* = 0.012), vaccination (*P <* 0.001), other healthy (*P* = 0.001) and post-operative (*P* = 0.003) consultations. Cats were prescribed antimicrobial agents significantly more frequently than dogs for respiratory (*P <* 0.001) and trauma (*P <* 0.001) consultations. Full results are presented in Table 1, Table 2.

**Table 1:** Canine antimicrobial agent prescription percentage (total, systemic and topical) by practitioner badged main presenting complaint calculated from total number of consultations for each category in a network of United Kingdom small animal veterinary premises.

Main presenting complaint	Dog						
	Number (%) of EHRs^a^	Total		Systemic		Topical	
		%	95% CI^b^	%	CI^b^	%	CI^b^
Pruritus	62,655 (6.8)	51.0	49.8–52.2	25.5	24.2–26.9	30.0	29.0–31.0
Respiratory	14,359 (1.6)	42.2	40.5–44.0	40.4	38.7–42.2	2.7	2.2–3.2
Gastroenteric	38,954 (4.2)	39.4	37.0–41.7	38.2	35.8–40.6	1.7	1.2–2.2
Trauma	58,033 (6.3)	26.7	25.5–27.9	21.3	20.3–22.4	6.2	5.8–6.6
Kidney disease	2607 (0.28)	29.1	26.6–31.7	26.8	24.3–29.3	3.0	2.2–3.7
Tumour	20,938 (2.3)	22.0	21.1–23.0	17.5	16.7–18.3	5.4	5.0–5.8
Other unwell	156,197 (17.0)	32.8	31.8–33.8	20.3	19.5–21.2	13.9	13.4–14.5
Post-operative	98,753 (10.8)	13.0	12.2–13.8	9.9	9.3–10.5	3.5	3.1–3.8
Vaccination	277,246 (30.2)	4.3	3.9–4.7	1.4	1.1–1.7	3.0	2.8–3.2
Other healthy	188,582 (20.6)	11.8	10.7–13.0	7.0	6.1–7.8	5.3	4.8–5.9

a Number (%) of electronic health records (EHRs). Relative occurrence of badged consultations as a frequency and as a percentage of total consultations.

b 95% Confidence interval.

**Table 2:** Feline antimicrobial agent prescription percentage (total, systemic and topical) by practitioner badged main presenting complaint calculated from total number of consultations for each category in a network of United Kingdom small animal veterinary premises.

Main presenting complaint	Cat						
	Number (%) of EHRs^a^	Total		Systemic		Topical	
		%	95% CI^b^	%	95% CI^b^	%	95% CI^b^
Pruritus	13,749 (3.9)	33.5	31.9–35.2	24.9	23.3–26.6	10.3	9.5–11.1
Respiratory	7681 (2.2)	52.0	49.8–54.3	59.9	47.6–52.2	5.3	4.6–5.9
Gastroenteric	11,206 (3.2)	29.8	27.4–31.8	28.9	26.7–31.1	1.0	0.7–1.4
Trauma	22,796 (6.5)	53.5	52.1–54.8	50.1	48.8–51.4	4.3	4.0–4.7
Kidney disease	4009 (1.1)	19.6	17.9–21.3	18.9	17.2–20.6	0.7	0.5–1.0
Tumour	5330 (1.5)	21.3	19.8–22.7	19.8	18.3–21.3	1.7	1.4–2.0
Other unwell	72,189 (20.5)	30.5	29.5–31.6	24.9	23.9–26.0	6.5	6.3–6.8
Post-operative	32,136 (9.1)	11.1	10.0–11.9	9.6	8.7–10.6	1.7	1.4–2.0
Vaccination	115,394 (32.6)	2.5	2.2–2.8	1.4	1.2–1.6	1.2	1.1–1.3
Other healthy	68,236 (19.4)	10.5	9.1–11.9	8.4	7.1–9.6	2.4	2.1–2.7

a Number (%) of electronic health records (EHRs). Relative occurrence of badged consultations as a frequency and as a percentage of total consultations.

b 95% Confidence interval.

### Level of antimicrobial agent prescription

A total of 218,700 canine and 71,089 feline AAPs were made from 215 practices (455 premises) in the UK.

#### Authorisation

For systemic AAP, 90.0% (95% CI 88.5–91.4) of canine and 92.9% (95% CI 91.7–94.1) of feline AAPs were species authorised, with 0.6% (95% CI 0.2–0.9) and 5.2% (95% CI 4.0–6.5) authorised in other veterinary species; of these, 8.2% (95% CI 7.0–9.4) and 1.7% (95% CI 1.4–2.1) were human authorised, 0.9% (95% CI 0.4–1.3) and 0.05% (95% CI 0.03–0.07) were dual generic and 0.4% (95% CI 0.1–0.6) and 0.04% (95% CI 0.00–0.09) were expired or of unknown authorisation, respectively. Metronidazole was the most frequently prescribed human authorised systemic antimicrobial agent in dogs (96.7% of human authorised systemic AAP, 95% CI 95.3–98.1) and cats (94.2%, 95% CI 92.1–96.3).

#### Class of antimicrobial agent

Clavulanic acid potentiated amoxicillin was the most frequently prescribed antimicrobial agent in dogs (28.6% of total AAP, 95% CI 27.4–29.8) and cefovecin was the most frequently prescribed antimicrobial agent in cats (36.2%, 95% CI 33.9–38.5) (Tables 3, 4 and 5). Fusidic acid was the most frequently prescribed topical antimicrobial agent in dogs (44.3% of topical AAP, 95% CI 43.1–45.4) and cats (55.1%, 95% CI 53.6–56.6).

**Table 3:** Percentage breakdown of canine antimicrobial agent prescriptions by antimicrobial agent class prescribed for total, systemic and topical prescriptions from a network of United Kingdom small animal veterinary premises.

Antimicrobial agent class	Total		Systemic		Topical	
	%	95% CI^a^	%	95% CI^a^	%	95% CI^a^
Aminoglycoside	12.0	11.4–12.6	0.1	0.0–0.2	29.1	28.0–30.2
Amphenicol	1.9	1.6–2.1	0.0	<0.00	4.5	3.9–5.2
Other antimicrobial agent^b^	7.2	6.6–7.8	0.0	<0.00	17.4	16.1–18.8
β-lactam	43.6	42.3–44.8	73.8	72.2–75.4	0.1	0.0–0.2
Fluoroquinolone	4.4	3.6–5.1	4.1	3.1–5.2	4.6	4.0–5.2
Fusidic acid	18.2	17.4–19.0	0.0	<0.00	44.3	43.1–45.4
Lincosamide	4.7	4.2–5.2	7.9	7.0–8.8	0.0	<0.00
Macrolide	0.2	0.0–0.3	0.3	0.0–0.6	0.0	<0.00
Nitroimidazole	4.7	4.0–5.4	8.0	6.7–9.2	0.0	<0.00
Nitroimidazole-macrolide	0.8	0.5–1.0	1.3	0.8–1.7	0.0	<0.00
Rifamycin	0.0	<0.00	0.0	<0.00	0.0	<0.00
Sulphonamide	1.5	1.1–1.9	2.5	1.9–3.2	0.0	<0.00
Tetracycline	1.2	1.0–1.3	2.0	1.7–2.2	0.0	0.00–0.01

a 95% Confidence interval.

b Consists of polymyxin b sulphate; mupirocin; novobiocin; thymol and bronopol.

**Table 4:** Percentage breakdown of feline antimicrobial agent prescriptions by antimicrobial agent class prescribed for total, systemic and topical prescriptions from a network of United Kingdom small animal veterinary premises.

Class of antimicrobial agent	Total		Systemic		Topical	
	%	95% CI^a^	%	95% CI^a^	%	95% CI^a^
Aminoglycoside	4.5	4.2–4.8	0.2	0.1–0.3	22.1	20.7–23.6
Amphenicol	1.3	1.1–1.5	0.0	<0.00	6.5	5.6–7.4
Other antimicrobial agent^b^	2.7	2.4–2.9	0.0	<0.00	13.5	12.4–14.6
β-lactam	70.8	69.3–72.3	87.9	86.1–89.7	0.3	0.0–0.6
Fluoroquinolone	3.0	1.7–4.3	3.1	1.6–4.7	2.5	2.0–3.0
Fusidic acid	10.8	10.2–11.3	0.0	<0.00	55.1	53.6–56.6
Lincosamide	4.1	3.5–4.7	5.2	4.4–5.9	0.0	<0.00
Macrolide	0.05	0.01–0.09	0.07	0.01–0.12	0.0	<0.00
Nitroimidazole	1.3	1.1–1.6	1.6	1.3–2.0	0.0	<0.00
Nitroimidazole-macrolide	0.4	0.2–0.5	0.5	0.3–0.7	0.0	<0.00
Rifamycin	0.0	<0.00	0.0^c^	<0.00	0.0	<0.00
Sulphonamide	0.05	0.03–0.07	0.06	0.03–0.09	0.0	<0.00
Tetracycline	1.1	1.0–1.3	1.4	1.2–1.6	0.0	<0.00

a 95% Confidence interval.

b Polymyxin b sulphate, mupirocin, novobiocin, thymol and bronopol.

c One recorded prescription of rifampicin for systemic administration (authorised for oral administration).

**Table 5:** Percentage breakdown of β-lactam antimicrobial agent prescription by species and β-lactam sub-categories as a percentage of total and systemic antimicrobial agent prescriptions from a network of small animal veterinary premises in the United Kingdom.

Class of antimicrobial agent	Total prescription				Systemic prescription			
	Dog		Cat		Dog		Cat	
	%	95% CI^a^	%	CI^a^	%	CI^a^	%	CI^a^
Amoxicillin	5.3	4.1–6.5	12.5	10.0–15.0	9.0	7.1–10.9	15.3	12.2–18.3
Other β-lactams^b^	0.4	0.0–0.8	0.07	0.01–0.13	0.5	0.0–1.3	0.02	0.00–0.05
First generation cephalosporin	8.4	7.8–9.0	0.4	0.3–0.5	14.2	13.2–15.3	0.5	0.4–0.6
Second generation cephalosporin	0.04	0.01–0.07	0.01	0.00–0.02	0.07	0.02–0.12	0.02	0.00–0.03
Third generation cephalosporin	0.9	0.7–1.0	36.2	33.9–38.5	1.5	1.3–1.8	45.1	42.1–48.2
Clavulanic acid potentiated amoxicillin	28.6	27.4–29.8	21.6	19.6–23.6	48.5	46.0–50.9	26.9	24.5–29.3
Penicillin	0.03	0.01–0.05	0.03	0.01–0.05	0.04	0.01–0.07	0.04	0.01–0.06
Total	43.6		70.8		73.8		87.9	

a 95% confidence interval.

b Ampicillin and cloxacillin.

#### Highest priority critically important antimicrobial agents

**Fig. 3:**
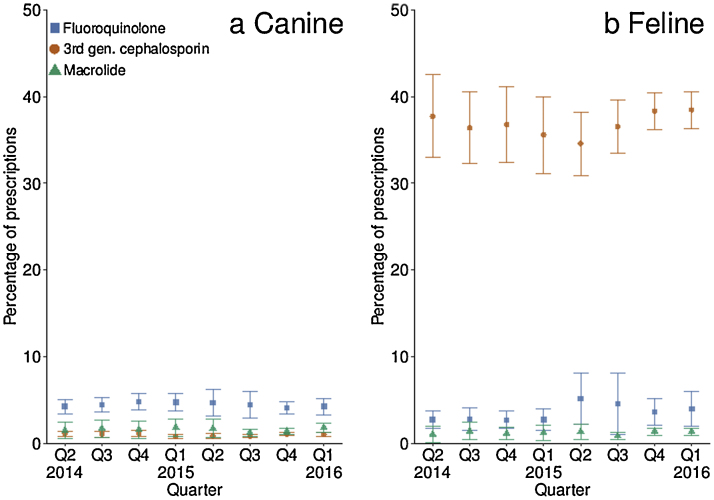
Comparison of (a) canine and (b) feline highest priority ‘critically important antimicrobial agent’ (HPCIA) prescription as a percentage of total antimicrobial agent prescriptions (95% confidence interval) by quarter (Q2 2014–Q1 2016).

Canine and feline HPCIA prescriptions were 5.4% (95% CI 4.6–6.1) and 39.2% (95% CI 36.8–41.7) of total AAPs respectively. On consideration of temporal trend, for canine HPCIA prescription, a significant positive cubic trend was noted (*P <* 0.001). Similarly, in cats, a significant positive linear trend was found (*P <* 0.001) (see Appendix: Supplementary Tables 3 and 4). The most frequently prescribed HPCIAs in dogs were fluoroquinolones and in cats was cefovecin, a third generation cephalosporin (Fig. 3).

#### Main presenting complaint

Total canine and feline AAPs summarised by MPCs are shown in Supplementary Tables 5 and 6 (see Appendix). Clavulanic acid potentiated amoxicillin was the most commonly prescribed antimicrobial agent in dogs for respiratory conditions, trauma, tumours and kidney disease, as well as other unwell, post-operative and other healthy MPCs. In cats, cefovecin was the most commonly prescribed antimicrobial agent for respiratory conditions, pruritus, trauma, tumours and kidney disease, as well as other unwell, post-operative and other healthy MPCs.

## Discussion

In this study, EHRs were used to describe AAP in a large population of companion animal veterinary premises. Quantitative differences in AAP were found between dogs and cats, and according to MPC. AAPC decreased significantly over the course of the study in this population of animals.

Broadly similar levels of total AAP were found in dogs and cats. However, when route of administration was considered, dogs were significantly more likely to be prescribed topical antimicrobial agents than cats, whereas cats were significantly more likely to be prescribed systemic antimicrobial agents than dogs. Such differences may reflect an increased prevalence of pruritus (and other dermatological diseases) in dogs compared to cats (Sánchez-Vizcaíno et al., 2016). They may also reflect the challenge of giving oral and topical medication to cats when compared to injectable antimicrobial agents (Burke et al., 2016).

Using data derived from EHRs, it was not possible to determine whether individual prescriptions were appropriate, nor whether the overall frequency of AAP in this population was appropriate. However, there was a significant reduction in canine and feline AAP within this population over the 2 years of the study. Whether this reflects the success of awareness campaigns is not known.^14^^,^^15^ It is possible that changes in AAP might reflect changes in other aspects of veterinary activity, such as vaccination. Furthermore, previous human AAP surveillance has noted short-term temporal variability that is not necessarily reflective of longer term patterns.^16^ As a consequence, there is a need to for ongoing monitoring of AAP.

Buckland et al. (2016) found that 25.2% of dogs and 20.6% of cats in the UK received systemic antimicrobial agents from 2012 to 2014. Whilst our results (2014–2016) were lower for dogs (19.6%), they were similar for cats (20.0%). In a smaller study conducted in the UK in 2010 (Radford et al., 2011), the proportion of consultations involving unhealthy animals where systemic antimicrobial agents were prescribed was 35.1% for dogs and 48.5% for cats. In our study, these values were lower (unhealthy dogs 24.1%, unhealthy cats 30.5%). It is unclear whether differences between these studies reflect a reduction in frequency of prescription of systemic antimicrobial agents, or are related to population differences or methods used to identify AAP.

Considerable variation in AAPs according to premise was identified in our study, as well as in the previous study by Radford et al. (2011). Premises that prescribed antimicrobial agents more frequently to dogs also tended to prescribe more frequently to cats. Such a correlation may be explained by geographical variation in risk (perceived or actual), either for AMR or for bacterial infections capable of infecting both species. Other complex factors, extending beyond the risk of antimicrobial agent responsive disease, can influence AAP decisions, such as clinical experience, perceived owner and/or pet compliance and practice policy (Hughes et al., 2012, Mateus et al., 2014).

It is not surprising that certain MPCs were more commonly associated with AAP, suggesting that practitioners believe that the risk of infection responsive to antimicrobial agents is higher in certain MPCs. Pruritus in dogs is frequently associated with bacterial pyoderma (Summers et al., 2014) and was associated with the most frequent use of topical antimicrobial agents in our study. However, acute respiratory disease in cats is generally considered to have a viral origin, although primary bacterial disease has been described and secondary bacterial infections can increase the severity of disease (Jacobs et al., 1993). Prescription of antimicrobial agents in feline trauma may reflect a high frequency of cat bite abscesses associated with this MPC (Radford et al., 2011, O’Neill et al., 2014b).

In dogs, clavulanic acid potentiated amoxicillin was the most frequently prescribed antimicrobial agent, as found in previous studies (Mateus et al., 2011, Radford et al., 2011, Buckland et al., 2016). In our study and that of Buckland et al. (2016), cefovecin was the most frequently prescribed antimicrobial agent in cats, in contrast to previous studies, where amoxicillin and clavulanic acid potentiated amoxicillin were more frequently prescribed (Mateus et al., 2011, Radford et al., 2011). This suggests that there has been a recent shift in choice of antimicrobial agents for cats. Prescription of cefovecin was common for MPCs associated with authorised indications for use, such as pruritus and kidney disease^17^ (Burke et al., 2016). However, cefovecin was also prescribed frequently in MPCs, such as respiratory and gastroenteric disease in cats, where there was no apparent indication for prescription by the datasheet^1^ or practice prescribing policy.^18^^,^^19^ It is also possible that relying on MPCs as declared by veterinary practitioners might fail to include other clinical conditions found during the same consultation. Collection and analysis of clinical free text present an opportunity to characterise each consultation based on clinical signs and duration, which would provide further information to support the rationale for any given prescription (Burke et al., 2016).

Although cefovecin is not authorised for human use, it is a third generation cephalosporin and is classified as an HPCIA.^20^^,^^21^ Relevant product information sheets state that cefovecin should be reserved for clinical conditions which have responded poorly, or are expected to respond poorly, to other classes of antimicrobial agents.^22^ In our study, it was not possible to determine to what extent the use of cefovecin is in compliance with these recommendations. A recent study showed that veterinary surgeons prescribing cefovecin rarely justified its use within the clinical narrative (Burke et al., 2016). Relative ease of administration and duration of action, together aiding compliance, may be important motivating factors for the use of cefovecin in veterinary practice. We noted considerable variation in prescription of cefovecin between premises, suggesting that there are differences in cat populations, presentations or justification for veterinary prescription. We further observed a slight increase in overall HPCIA prescription in dogs and cats throughout the study, and that many of the most commonly prescribed antimicrobial agents in both species are considered to be critically important.^23^

Whilst such large volumes of data provide new insights into AAP, the nature of these data have their own inherent limitations. Quantification of AAP relies on practitioners charging for antimicrobial agents through their practice management software, which means that any antimicrobial agents not charged for will be missed. The SAVSNET population of practices is recruited on the basis of convenience and so cannot necessarily be considered to be representative of the wider UK population. In order to fully place findings in context, there is a need for in depth analysis of the animal populations monitored. The use of the MPC function allows all consultations to be coded in real time; variations in individual interpretation of the MPC case definition are possible.

## Conclusions

AAP frequency decreased from 2014 to 2016 in this population of dogs and cats in the UK. Additionally, some MPCs were more likely to be associated with AAP than others, both within and between the two species. There is considerable variability in AAP amongst different premises and there is a need to understand factors that influence AAP at the individual animal, owner and premise level, particularly for HPCIAs. To aid responsible use, SAVSNET provides a mechanism for participating practices to benchmark their prescription against anonymised peers via an online portal. This and other studies are now providing the valuable tools and data that the profession needs to ensure antimicrobial agents are used responsibly.

## Conflicts of interest statement

None of the authors of this paper have a financial or personal relationship with other people or organisations that could inappropriately influence or bias the content of this paper.

## Footnotes

1 See: http://www.ema.europa.eu/docs/en_GB/document_library/Scientific_guideline/2015/01/WC500181642.pdf. (Accessed 15 July 2016).

2 See: https://www.gov.uk/government/publications/uk-one-health-report-antibiotics-use-in-humans-and-animals. (Accessed 15 July 2016).

3 See: http://amr-review.org/home. (Accessed 15 July 2016).

4 See: http://apps.who.int/iris/bitstream/10665/112642/1/9789241564748_eng.pdf. (Accessed 15 July 2016).

5 See: http://www.bsava.com/Resources/PROTECT.aspx. (Accessed 4 October 2016).

6 See: http://www.fecava.org/content/guidelines-policies. (Accessed 15 July 2016).

7 See: http://fingertips.phe.org.uk. (Accessed 15 July 2016).

8 See: https://www.gov.uk/government/publications/veterinary-antimicrobial-resistance-and-sales-surveillance-2014. (Accessed 15 July 2016).

9 See: http://www.who.int/foodsafety/publications/antimicrobials-fourth/en/. (Accessed 13 February 2017).

10 See: http://cran.r-project.org/package=aod. (Accessed 11 October 2016).

11 See: http://www.ats.ucla.edu/stat/r/library/contrast_coding.htm#ORTHOGONAL. (Accessed 11 October 2016).

12 See: http://www.ats.ucla.edu/stat/r/library/contrast_coding.htm#backward. (Accessed 11 October 2016).

13 See: http://www.R-project.org/. (Accessed 23 November 2016).

14 See: http://www.fecava.org/content/guidelines-policies. (Accessed 15 July 2016).

15 See: http://www.bsava.com/Resources/PROTECT.aspx. (Accessed 4 October 2016).

16 See: http://ecdc.europa.eu/en/publications/Publications/antimicrobial-consumption-europe-esac-net-2012.pdf. (Accessed 26 January 2017).

17 See: http://www.ema.europa.eu/docs/en_GB/document_library/EPAR_-_Product_Information/veterinary/000098/WC500062067.pdf. (Accessed 12 December 2016).

18 See: http://www.fecava.org/content/guidelines-policies. (Accessed 15 July 2016).

19 See: http://www.bsava.com/Resources/PROTECT.aspx. (Accessed 4 October 2016).

20 See: http://www.who.int/foodsafety/publications/antimicrobials-fourth/en/. (Accessed 13 February 2017).

21 See: http://www.noah.co.uk/wp-content/uploads/2016/12/NOAH-briefing-on-CIAs-07122016.pdf. (Accessed 14 February 2017).

22 See: http://www.ema.europa.eu/docs/en_GB/document_library/EPAR_-_Product_Information/veterinary/000098/WC500062067.pdf. (Accessed 12 December 2016).

23 See: http://www.who.int/foodsafety/publications/antimicrobials-fourth/en/. (Accessed 13 February 2017).
