# Supplementary material for: Patterns of antimicrobial agent prescription in a sentinel population of canine and feline veterinary practices in the United Kingdom
Source: Vet J. 2017 Jun;224:18–24. doi: 10.1016/j.tvjl.2017.03.010 (PMC5518771; doi:10.1016/j.tvjl.2017.03.010)
Supplement: Supplementary file 1 [file mmc1.docx]

**Appendix: Supplementary material**

**Supplementary Table 1**

Definitions of main presenting complaints as provided to participating veterinary surgeons as a hover box in the United Kingdom Small Animal Veterinary Surveillance Network (SAVSNET) interactive window which appears at the end of each consultation.

| Main presenting complaint | Group | Definition |
| --- | --- | --- |
| Gastroenteric | Unhealthy | Signs including but not limited to diarrhoea, vomiting, weight loss, poor appetite |
| Pruritus | Unhealthy | Signs including but not limited to itching, scratching, pruritic otitis, chewing, licking, rubbing |
| Respiratory | Unhealthy | Signs associated with conditions affecting the upper and / or lower respiratory tract |
| Tumour | Unhealthy | Any suspected or confirmed benign or malignant neoplastic condition |
| Trauma | Unhealthy | Animal suffering a trauma and / or a physical injury |
| Kidney disease | Unhealthy | Signs including but not limited to polydipsia, polyuria, vomiting where kidney disease is a differential |
| Other unwell | Unhealthy | Signs that do not fit in other unwell animal categories including behaviour problems |
| Post-operative | Post-operative | If the animal has presented for post-operative care |
| Vaccination | Healthy | If the animal was booked in for a vaccination and was vaccinated |
| Other healthy | Healthy | Healthy animal presented for other reasons that do not fit in the vaccination or in the post-operative check categories |

**Supplementary Table 2**

Orthogonal polynomial contrast coding values (to four decimal places) utilised for construction of eight mixed effects binomial regression models, modelling the probability of canine and feline total, systemic, topical and highest priority critically important antimicrobial agent (HPCIA) antimicrobial agent prescription in a network of United Kingdom small animal veterinary premises.

| Linear | Quadratic | Cubic | Quartic | Quintic | Sextic | Septic |
| --- | --- | --- | --- | --- | --- | --- |
| -0.5401 | 0.5401 | -0.4308 | 0.2820 | -0.1498 | 0.0615 | -0.0171 |
| -0.3858 | 0.0772 | 0.3077 | -0.5238 | 0.4922 | -0.3077 | 0.1195 |
| -0.2315 | -0.2315 | 0.4308 | -0.1209 | -0.3638 | 0.5539 | -0.3585 |
| -0.0772 | -0.3858 | 0.1846 | 0.3626 | -0.3210 | -0.3077 | 0.5974 |
| 0.0772 | -0.3858 | -0.1846 | 0.3626 | 0.3210 | -0.3077 | -0.5974 |
| 0.2315 | -0.2315 | -0.4308 | -0.1209 | 0.3638 | 0.5539 | 0.3585 |
| 0.3858 | 0.0772 | -0.3077 | -0.5238 | -0.4922 | -0.3077 | -0.1195 |
| 0.5401 | 0.5401 | 0.4308 | 0.2820 | 0.1498 | 0.0615 | 0.0171 |

The models incorporate practice and premise as random effects and the ordinal variable quarter as a fixed effect. The variable quarter (Q2 2014 to Q1 2016) was codified using the orthogonal polynomial coding system.

**Supplementary Table 3**

Parameter estimates from eight fitted mixed effects binomial regression models, modelling the probability of canine and feline total, systemic, topical and HPCIA ^d^ antimicrobial agent prescription in a network of United Kingdom small animal veterinary premises.

| Species | Model | Random effect | Variance | SD ^a^ | Fixed effect | β | SE ^b^ | *P* |
| --- | --- | --- | --- | --- | --- | --- | --- | --- |
| Canine | Total | Practice | 0.036 | 0.189 | Intercept | -1.702 | 0.016 | <0.0001 |
|  |  | Premise | 0.018 | 0.133 | Quarter - linear fit | -0.127 | 0.011 | <0.0001 |
|  |  |  |  |  | Quarter - quadratic fit | 0.005 | 0.010 | 0.6106 |
|  |  |  |  |  | Quarter - cubic fit | 0.012 | 0.009 | 0.1760 |
|  |  |  |  |  | Quarter - quartic fit | -0.068 | 0.009 | <0.0001 |
|  |  |  |  |  | Quarter - quintic fit | -0.027 | 0.009 | 0.0013 |
|  |  |  |  |  | Quarter - sextic fit | 0.013 | 0.009 | 0.1275 |
|  |  |  |  |  | Quarter - septic fit | 0.012 | 0.010 | 0.2474 |
| Canine | Systemic | Practice | 0.055 | 0.233 | Intercept | -2.160 | 0.020 | <0.0001 |
|  |  | Premise | 0.039 | 0.198 | Quarter - linear fit | -0.186 | 0.014 | <0.0001 |
|  |  |  |  |  | Quarter - quadratic fit | -0.015 | 0.012 | 0.2178 |
|  |  |  |  |  | Quarter - cubic fit | 0.036 | 0.010 | 0.0001 |
|  |  |  |  |  | Quarter - quartic fit | -0.077 | 0.010 | <0.0001 |
|  |  |  |  |  | Quarter - quintic fit | -0.039 | 0.010 | 0.0001 |
|  |  |  |  |  | Quarter - sextic fit | 0.020 | 0.011 | 0.0572 |
|  |  |  |  |  | Quarter - septic fit | 0.020 | 0.012 | 0.1008 |
| Canine | Topical | Practice | 0.034 | 0.183 | Intercept | -2.622 | 0.016 | <0.0001 |
|  |  | Premise | 0.014 | 0.119 | Quarter - linear fit | -0.060 | 0.017 | 0.0003 |
|  |  |  |  | | Quarter - quadratic fit | 0.037 | 0.015 | 0.0147 |
|  |  |  |  |  | Quarter - cubic fit | -0.030 | 0.013 | 0.0213 |
|  |  |  |  |  | Quarter - quartic fit | -0.058 | 0.013 | <0.0001 |
|  |  |  |  | | Quarter - quintic fit | -0.006 | 0.013 | 0.6458 |
|  |  |  |  | | Quarter - sextic fit | -0.001 | 0.014 | 0.9293 |
|  |  |  |  | | Quarter - septic fit | -0.004 | 0.016 | 0.8217 |
| Canine | HPCIA ^d^ | Practice | 0.201 | 0.449 | Intercept | -3.162 | 0.044 | <0.0001 |
|  |  | Premise | 0.175 | 0.418 | Quarter - linear fit | -0.063 | 0.039 | 0.1064 |
|  |  |  |  | | Quarter - quadratic fit | -0.051 | 0.034 | 0.1366 |
|  |  |  |  |  | Quarter - cubic fit | 0.130 | 0.030 | <0.0001 |
|  |  |  |  | | Quarter - quartic fit | 0.029 | 0.030 | 0.3400 |
|  |  |  |  | | Quarter - quintic fit | -0.066 | 0.030 | 0.0271 |
|  |  |  |  | | Quarter - sextic fit | -0.045 | 0.031 | 0.1143 |
|  |  |  |  | | Quarter - septic fit | -0.004 | 0.035 | 0.9071 |
| Feline | Total | Practice | 0.061 | 0.247 | Intercept | -1.752 | 0.021 | <0.0001 |
|  |  | Premise | 0.025 | 0.157 | Quarter - linear fit | -0.195 | 0.019 | <0.0001 |
|  |  |  |  |  | Quarter - quadratic fit | 0.029 | 0.017 | 0.0862 |
|  |  |  |  |  | Quarter - cubic fit | 0.010 | 0.014 | 0.4980 |
|  |  |  |  |  | Quarter - quartic fit | 0.021 | 0.014 | 0.1506 |
|  |  |  |  |  | Quarter - quintic fit | -0.021 | 0.014 | 0.1320 |
|  |  |  |  |  | Quarter - sextic fit | -0.027 | 0.015 | 0.0631 |
|  |  |  |  |  | Quarter - septic fit | -0.002 | 0.017 | 0.9254 |
| Feline | Systemic | Practice | 0.078 | 0.279 | Intercept | -1.934 | 0.024 | <0.0001 |
|  |  | Premise | 0.039 | 0.198 | Quarter - linear fit | -0.206 | 0.020 | <0.0001 |
|  |  |  |  |  | Quarter - quadratic fit | 0.014 | 0.018 | 0.4450 |
|  |  |  |  |  | Quarter - cubic fit | 0.026 | 0.015 | 0.0933 |
|  |  |  |  |  | Quarter - quartic fit | 0.025 | 0.015 | 0.1067 |
|  |  |  |  |  | Quarter - quintic fit | -0.029 | 0.015 | 0.0529 |
|  |  |  |  |  | Quarter - sextic fit | -0.031 | 0.016 | 0.0457 |
|  |  |  |  |  | Quarter - septic fit | -0.007 | 0.018 | 0.7015 |
| Feline | Topical | Practice | 0.028 | 0.168 | Intercept | -3.435 | 0.021 | <0.0001 |
|  |  | Premise | 0.024 | 0.155 | Quarter - linear fit | -0.163 | 0.037 | <0.0001 |
|  |  |  |  |  | Quarter - quadratic fit | 0.113 | 0.036 | 0.0018 |
|  |  |  |  |  | Quarter - cubic fit | -0.073 | 0.032 | 0.0209 |
|  |  |  |  |  | Quarter - quartic fit | -0.020 | 0.032 | 0.5307 |
|  |  |  |  |  | Quarter - quintic fit | 0.011 | 0.031 | 0.7186 |
|  |  |  |  |  | Quarter - sextic fit | -0.017 | 0.033 | 0.5958 |
|  |  |  |  |  | Quarter - septic fit | 0.037 | 0.038 | 0.3324 |
| Feline | HPCIA ^c^ | Practice | 0.141 | 0.376 | Intercept | -1.009 | 0.032 | <0.0001 |
|  |  | Premise | 0.037 | 0.192 | Quarter - linear fit | 0.106 | 0.031 | 0.0007 |
|  |  |  |  |  | Quarter - quadratic fit | -0.004 | 0.028 | 0.8866 |
|  |  |  |  |  | Quarter - cubic fit | -0.015 | 0.024 | 0.5114 |
|  |  |  |  |  | Quarter - quartic fit | 0.009 | 0.024 | 0.6955 |
|  |  |  |  |  | Quarter - quintic fit | -0.005 | 0.023 | 0.8173 |
|  |  |  |  |  | Quarter - sextic fit | 0.015 | 0.024 | 0.5339 |
|  |  |  |  |  | Quarter - septic fit | -0.021 | 0.028 | 0.4597 |

The models incorporate practice and premise as random effects and the ordinal variable quarter as a fixed effect. The variable quarter (Q2 2014 - Q1 2016) was codified using an orthogonal polynomial coding system.

^a^ Standard deviation.

^b^ Standard error.

^c^ Highest priority critically important antimicrobial agent.

**Table 4**

Parameter estimates from eight fitted mixed effects binomial regression models, modelling the probability of canine and feline total, systemic, topical and highest priority critically important antimicrobial agent (HPCIA) prescription in a network of United Kingdom small animal veterinary premises.

| Species | Model | Random effect | Variance | SD ^a^ | Fixed effect | β | SE ^b^ | OR ^c^ | Lower 95% CI ^d^ | Upper 95% CI ^d^ | *P* |
| --- | --- | --- | --- | --- | --- | --- | --- | --- | --- | --- | --- |
| Canine | Total | Practice | 0.036 | 0.190 | Intercept | -1.704 | 0.016 | 0.182 | 0.177 | 0.188 | <0.001 |
|  |  | Premise | 0.017 | 0.132 | 2014 Q3 cf. 2014 Q2 | -0.059 | 0.012 | 0.943 | 0.921 | 0.965 | <0.001 |
|  |  |  |  | | 2014 Q4 cf. 2014 Q3 | 0.041 | 0.010 | 1.041 | 1.021 | 1.061 | <0.001 |
|  |  |  |  |  | 2015 Q1 cf. 2014 Q4 | -0.018 | 0.010 | 0.982 | 0.963 | 1.003 | 0.087 |
|  |  |  |  |  | 2015 Q2 cf. 2015 Q1 | -0.073 | 0.011 | 0.930 | 0.910 | 0.949 | <0.001 |
|  |  |  |  |  | 2015 Q3 cf. 2015 Q2 | -0.040 | 0.008 | 0.961 | 0.946 | 0.976 | <0.001 |
|  |  |  |  |  | 2015 Q4 cf. 2015 Q3 | -0.023 | 0.008 | 0.978 | 0.963 | 0.992 | 0.003 |
|  |  |  |  |  | 2016 Q1 cf. 2015 Q4 | -0.098 | 0.008 | 0.907 | 0.893 | 0.921 | <0.001 |
| Canine | Systemic | Practice | 0.055 | 0.234 | Intercept | -2.159 | 0.020 | 0.115 | 0.111 | 0.120 | <0.001 |
|  |  | Premise | 0.039 | 0.198 | 2014 Q3 cf. 2014 Q2 | -0.071 | 0.014 | 0.931 | 0.906 | 0.958 | <0.001 |
|  |  |  |  | | 2014 Q4 cf. 2014 Q3 | 0.068 | 0.012 | 1.071 | 1.047 | 1.095 | <0.001 |
|  |  |  |  |  | 2015 Q1 cf. 2014 Q4 | -0.004 | 0.012 | 0.996 | 0.972 | 1.020 | 0.736 |
|  |  |  |  |  | 2015 Q2 cf. 2015 Q1 | -0.094 | 0.013 | 0.910 | 0.887 | 0.933 | <0.001 |
|  |  |  |  |  | 2015 Q3 cf. 2015 Q2 | -0.062 | 0.010 | 0.940 | 0.922 | 0.958 | <0.001 |
|  |  |  |  |  | 2015 Q4 cf. 2015 Q3 | -0.054 | 0.009 | 0.948 | 0.931 | 0.965 | <0.001 |
|  |  |  |  |  | 2016 Q1 cf. 2015 Q4 | -0.140 | 0.009 | 0.869 | 0.854 | 0.885 | <0.001 |
| Canine | Topical | Practice | 0.034 | 0.183 | Intercept | -2.622 | 0.016 | 0.073 | 0.070 | 0.075 | <0.001 |
|  |  | Premise | 0.014 | 0.119 | 2014 Q3 cf. 2014 Q2 | -0.057 | 0.018 | 0.945 | 0.912 | 0.979 | 0.002 |
|  |  |  |  | | 2014 Q4 cf. 2014 Q3 | -0.005 | 0.015 | 0.995 | 0.966 | 1.025 | 0.758 |
|  |  |  |  |  | 2015 Q1 cf. 2014 Q4 | -0.043 | 0.015 | 0.955 | 0.929 | 0.989 | 0.008 |
|  |  |  |  |  | 2015 Q2 cf. 2015 Q1 | -0.041 | 0.016 | 0.960 | 0.930 | 0.991 | 0.012 |
|  |  |  |  |  | 2015 Q3 cf. 2015 Q2 | -0.014 | 0.012 | 0.987 | 0.963 | 1.011 | 0.272 |
|  |  |  |  |  | 2015 Q4 cf. 2015 Q3 | 0.016 | 0.011 | 1.016 | 0.994 | 1.039 | 0.159 |
|  |  |  |  |  | 2016 Q1 cf. 2015 Q4 | -0.050 | 0.011 | 0.951 | 0.930 | 0.973 | <0.001 |
| Canine | HPCIA | Practice | 0.201 | 0.449 | Intercept | -3.162 | 0.044 | 0.042 | 0.039 | 0.046 | <0.001 |
|  |  | Premise | 0.175 | 0.418 | 2014 Q3 cf. 2014 Q2 | 0.039 | 0.041 | 1.040 | 0.959 | 1.127 | 0.344 |
|  |  |  |  | | 2014 Q4 cf. 2014 Q3 | 0.069 | 0.033 | 1.071 | 1.004 | 1.143 | 0.038 |
|  |  |  |  |  | 2015 Q1 cf. 2014 Q4 | 0.081 | 0.036 | 1.084 | 1.011 | 1.163 | 0.024 |
|  |  |  |  |  | 2015 Q2 cf. 2015 Q1 | -0.014 | 0.037 | 0.986 | 0.917 | 1.060 | 0.702 |
|  |  |  |  |  | 2015 Q3 cf. 2015 Q2 | -0.123 | 0.029 | 0.884 | 0.835 | 0.936 | <0.001 |
|  |  |  |  |  | 2015 Q4 cf. 2015 Q3 | -0.047 | 0.027 | 0.954 | 0.905 | 1.006 | 0.081 |
|  |  |  |  |  | 2016 Q1 cf. 2015 Q4 | -0.021 | 0.027 | 0.979 | 0.928 | 1.033 | 0.446 |
| Feline | Total | Practice | 0.061 | 0.247 | Intercept | -1.752 | 0.021 | 0.173 | 0.166 | 0.181 | <0.001 |
|  |  | Premise | 0.025 | 0.157 | 2014 Q3 cf. 2014 Q2 | -0.142 | 0.021 | 0.868 | 0.833 | 0.904 | <0.001 |
|  |  |  |  | | 2014 Q4 cf. 2014 Q3 | 0.028 | 0.016 | 1.028 | 0.997 | 1.061 | 0.078 |
|  |  |  |  |  | 2015 Q1 cf. 2014 Q4 | 0.022 | 0.017 | 1.022 | 0.989 | 1.057 | 0.194 |
|  |  |  |  |  | 2015 Q2 cf. 2015 Q1 | -0.024 | 0.018 | 0.977 | 0.943 | 1.012 | 0.189 |
|  |  |  |  |  | 2015 Q3 cf. 2015 Q2 | -0.087 | 0.014 | 0.916 | 0.892 | 0.941 | <0.001 |
|  |  |  |  |  | 2015 Q4 cf. 2015 Q3 | -0.073 | 0.013 | 0.929 | 0.906 | 0.952 | <0.001 |
|  |  |  |  |  | 2016 Q1 cf. 2015 Q4 | -0.090 | 0.013 | 0.914 | 0.891 | 0.937 | <0.001 |
| Feline | Systemic | Practice | 0.078 | 0.279 | Intercept | -1.934 | 0.024 | 0.145 | 0.138 | 0.152 | <0.001 |
|  |  | Premise | 0.039 | 0.198 | 2014 Q3 cf. 2014 Q2 | -0.134 | 0.022 | 0.875 | 0.837 | 0.914 | <0.001 |
|  |  |  |  | | 2014 Q4 cf. 2014 Q3 | 0.042 | 0.017 | 1.042 | 1.008 | 1.077 | 0.014 |
|  |  |  |  |  | 2015 Q1 cf. 2014 Q4 | 0.032 | 0.018 | 1.033 | 0.997 | 1.070 | 0.072 |
|  |  |  |  |  | 2015 Q2 cf. 2015 Q1 | -0.020 | 0.019 | 0.980 | 0.944 | 1.018 | 0.304 |
|  |  |  |  |  | 2015 Q3 cf. 2015 Q2 | -0.102 | 0.015 | 0.903 | 0.877 | 0.929 | <0.001 |
|  |  |  |  |  | 2015 Q4 cf. 2015 Q3 | -0.082 | 0.014 | 0.922 | 0.897 | 0.946 | <0.001 |
|  |  |  |  |  | 2016 Q1 cf. 2015 Q4 | -0.099 | 0.014 | 0.906 | 0.881 | 0.931 | <0.001 |
| Feline | Topical | Practice | 0.028 | 0.168 | Intercept | -3.435 | 0.021 | 0.032 | 0.031 | 0.034 | <0.001 |
|  |  | Premise | 0.024 | 0.155 | 2014 Q3 cf. 2014 Q2 | -0.196 | 0.043 | 0.822 | 0.755 | 0.895 | <0.001 |
|  |  |  |  | | 2014 Q4 cf. 2014 Q3 | -0.047 | 0.036 | 0.954 | 0.889 | 1.024 | 0.191 |
|  |  |  |  |  | 2015 Q1 cf. 2014 Q4 | -0.031 | 0.036 | 0.970 | 0.889 | 1.024 | 0.419 |
|  |  |  |  |  | 2015 Q2 cf. 2015 Q1 | -0.066 | 0.041 | 0.936 | 0.865 | 1.014 | 0.106 |
|  |  |  |  |  | 2015 Q3 cf. 2015 Q2 | -0.025 | 0.029 | 0.975 | 0.921 | 1.032 | 0.387 |
|  |  |  |  |  | 2015 Q4 cf. 2015 Q3 | -0.029 | 0.026 | 0.972 | 0.924 | 1.022 | 0.263 |
|  |  |  |  |  | 2016 Q1 cf. 2015 Q4 | -0.066 | 0.026 | 0.937 | 0.890 | 0.985 | 0.011 |
| Feline | HPCIA | Practice | 0.141 | 0.376 | Intercept | -1.009 | 0.032 | 0.365 | 0.343 | 0.388 | <0.001 |
|  |  | Premise | 0.037 | 0.192 | 2014 Q3 cf. 2014 Q2 | 0.055 | 0.033 | 1.057 | 0.990 | 1.128 | 0.099 |
|  |  |  |  | | 2014 Q4 cf. 2014 Q3 | -0.002 | 0.026 | 0.998 | 0.948 | 1.128 | 0.934 |
|  |  |  |  |  | 2015 Q1 cf. 2014 Q4 | -0.010 | 0.028 | 0.990 | 0.937 | 1.047 | 0.732 |
|  |  |  |  |  | 2015 Q2 cf. 2015 Q1 | 0.034 | 0.030 | 1.034 | 0.975 | 1.097 | 0.263 |
|  |  |  |  |  | 2015 Q3 cf. 2015 Q2 | 0.042 | 0.032 | 1.043 | 0.997 | 1.090 | 0.067 |
|  |  |  |  |  | 2015 Q4 cf. 2015 Q3 | 0.053 | 0.021 | 1.054 | 1.012 | 1.098 | 0.011 |
|  |  |  |  |  | 2016 Q1 cf. 2015 Q4 | 0.063 | 0.021 | 1.065 | 1.021 | 1.110 | 0.003 |

The models incorporate practice and premise as random effects and the ordinal variable quarter as a fixed effect. The variable quarter was codified using a backwards difference contrast coding system.

^a^ Standard deviation.

^b^ Standard error.

^c^ Odds ratio.

^d^ 95% Confidence interval.

**Table 5**

Canine antimicrobial agent prescription (systemic and topical) by class as percentage of total prescriptions for each main presenting complaint in a network of United Kingdom small animal veterinary premises (data collected from 1 April 2014 to 31 March 2016).

| Antimicrobial agent class | Gastroenteric | | Respiratory | | Pruritus | | Trauma | | Tumour | | Kidney disease | | Other unwell | | Post-operative | | Vaccination | | Other healthy | |
| --- | --- | --- | --- | --- | --- | --- | --- | --- | --- | --- | --- | --- | --- | --- | --- | --- | --- | --- | --- | --- |
|  | % | 95% CI ^a^ | % | 95% CI ^a^ | % | 95% CI ^a^ | % | 95% CI ^a^ | % | 95% CI ^a^ | % | 95% CI ^a^ | % | 95% CI ^a^ | % | 95% CI ^a^ | % | 95% CI ^a^ | % | 95% CI ^a^ |
| Aminoglycoside | 1.2 | 1.0-1.4 | 1.9 | 1.3-2.6 | 18.8 | 17.8-19.8 | 4.4 | 3.9-5.0 | 3.7 | 3.2-4.3 | 2.9 | 1.8-3.9 | 12.4 | 11.7-13.0 | 7.1 | 6.2-7.9 | 23.4 | 21.4-25.3 | 14.3 | 12.4-16.1 |
| Amphenicol | 0.09 | 0.04-0.13 | 0.3 | 0.1-0.5 | 2.1 | 1.7-2.6 | 1.5 | 1.2-1.8 | 0.4 | 0.2-0.6 | 0.6 | 0.1-1.0 | 2.5 | 2.2-2.9 | 1.8 | 1.4-2.2 | 1.7 | 1.1-2.4 | 2.1 | 1.7-2.6 |
| Other agent ^b^ | 1.2 | 0.1-2.3 | 1.3 | 0.8-1.9 | 10.2 | 9.2-11.3 | 2.2 | 1.9-2.6 | 2.6 | 2.1-3.2 | 2.4 | 1.3-3.5 | 7.6 | 7.0-8.1 | 4.5 | 3.8-5.3 | 13.5 | 12.0-15.0 | 9.5 | 7.5-11.4 |
| β-lactam (total) | 47.3 | 43.0-51.6 | 62.0 | 58.8-65.2 | 34.2 | 32.4-36.1 | 68.0 | 66.3-69.8 | 61.8 | 59.5-64.1 | 77.3 | 73.6-81.0 | 42.1 | 40.9-43.4 | 56.9 | 54.7-59.1 | 18.8 | 16.3-21.3 | 39.6 | 36.2-42.9 |
| Amoxicillin | 15.6 | 12.5-18.8 | 5.9 | 3.9-7.9 | 1.3 | 0.7-1.9 | 9.0 | 6.6-11.4 | 5.9 | 4.0-7.9 | 5.9 | 3.7-8.1 | 4.4 | 3.3-5.4 | 5.1 | 3.9-6.2 | 1.7 | 1.0-2.4 | 6.0 | 3.9-8.2 |
| Other β-lactam ^c^ | 0.09 | 0.03-0.16 | 0.3 | 0.0-0.5 | 0.2 | 0.0-0.4 | 0.5 | 0.0-1.2 | 0.7 | 0.0-1.7 | 0.7 | 0.0-1.6 | 0.3 | 0.0-0.6 | 0.3 | 0.0-0.8 | 0.1 | 0.0-0.3 | 0.8 | 0.0-2.1 |
| First generation cephalosporin | 0.6 | 0.3-0.9 | 2.9 | 2.2-3.7 | 20.3 | 18.7-21.8 | 7.4 | 6.4-8.5 | 9.7 | 8.2-11.3 | 4.9 | 3.0-6.9 | 5.8 | 5.3-6.3 | 7.4 | 6.4-8.5 | 4.7 | 4.0-5.4 | 6.3 | 5.3-7.1 |
| Second generation cephalosporin | 0.04 | 0.01-0.07 | 0.03 | 0.00-0.07 | 0.00 | 0.00-0.01 | 0.2 | 0.0-0.5 | 0.04 | 0.00-0.09 | 0.0 | <0.00 | 0.03 | 0.01-0.05 | 0.08 | 0.00-0.16 | 0.0 | <0.00 | 0.03 | 0.00-0.06 |
| Third generation cephalosporin | 0.5 | 0.4-0.7 | 1.3 | 1.0-1.7 | 1.2 | 0.9-1.4 | 0.7 | 0.5-0.9 | 1.1 | 0.7-1.5 | 2.4 | 1.3-3.5 | 1.0 | 0.8-1.2 | 0.9 | 0.6-1.1 | 0.4 | 0.3-0.6 | 0.7 | 0.5-0.9 |
| Clavulanic acid potentiated amoxicillin | 30.2 | 26.6-33.9 | 51.7 | 47.9-55.4 | 11.4 | 10.3-12.4 | 50.2 | 47.2-53.1 | 44.2 | 41.5-46.9 | 63.3 | 58.4-68.2 | 30.6 | 29.6-31.7 | 43.1 | 40.8-45.5 | 11.9 | 9.9-13.8 | 25.8 | 23.1-28.5 |
| Penicillin | 0.1 | 0.0-0.2 | 0.0 | <0.00 | 0.0 | <0.00 | 0.02 | 0.00-0.03 | 0.05 | 0.00-0.11 | 0.1 | 0.0-0.3 | 0.02 | 0.01-0.04 | 0.03 | 0.00-0.09 | 0.01 | 0.00-0.02 | 0.06 | 0.01-0.11 |
| Fluoroquinolone | 4.3 | 0.7-7.9 | 6.2 | 4.5-8.0 | 4.5 | 3.7-5.2 | 2.3 | 1.9-2.6 | 2.7 | 2.1-3.4 | 5.7 | 3.1-8.3 | 5.1 | 4.4-5.8 | 4.8 | 4.2-5.5 | 3.1 | 2.4-3.9 | 3.7 | 3.0-4.4 |
| Fusidic acid | 1.9 | 1.7-2.2 | 3.1 | 2.8-3.7 | 25.4 | 24.1-26.7 | 12.6 | 11.6-13.6 | 16.5 | 15.2-17.8 | 5.1 | 3.5-6.7 | 18.9 | 18.0-19.7 | 13.2 | 12.3-14.2 | 32.3 | 30.0-34.7 | 19.7 | 18.4-21.1 |
| Lincosamide | 1.2 | 0.8-1.7 | 1.2 | 0.8-1.5 | 2.9 | 2.3-3.4 | 5.8 | 4.8-6.8 | 8.2 | 6.9-9.8 | 1.4 | 0.6-2.3 | 6.4 | 5.8-7.0 | 5.5 | 4.5-6.3 | 3.2 | 2.7-3.8 | 5.5 | 4.5-6.5 |
| Macrolide | 1.1 | 0.0-2.3 | 0.0 | <0.00 | 0.01 | 0.00-0.02 | 0.02 | 0.00-0.05 | 0.04 | 0.00-0.11 | 0.0 | <0.00 | 0.04 | 0.00-0.09 | 0.06 | 0.00-0.14 | 0.2 | 0.0-0.3 | 0.2 | 0.0-0.6 |
| Nitroimidazole | 33.5 | 28.4-38.5 | 0.8 | 0.5-1.0 | 0.3 | 0.2-0.3 | 1.3 | 0.6-2.0 | 1.6 | 0.5-2.7 | 1.2 | 0.5-2.0 | 2.6 | 2.2-3.0 | 3.6 | 2.9-4.4 | 2.1 | 1.4-2.8 | 2.9 | 2.3-3.5 |
| Nitroimidazole-macrolide | 5.0 | 3.0-7.1 | 0.2 | 0.0-0.4 | 0.06 | 0.03-0.10 | 0.13 | 0.05-0.20 | 0.3 | 0.1-0.4 | 0.2 | 0.0-0.7 | 0.4 | 0.3-0.6 | 0.4 | 0.2-0.6 | 0.3 | 0.2-0.4 | 0.9 | 0.3-1.4 |
| Rifamycin | 0.0 | <0.00 | 0.0 | <0.00 | 0.0 | <0.00 | 0.0 | <0.00 | 0.0 | <0.00 | 0.0 | <0.00 | 0.0 | <0.00 | 0.0 | <0.00 | 0.0 | <0.00 | 0.0 | <0.00 |
| Sulphonamide | 2.3 | 0.1-4.5 | 6.1 | 3.9-8.1 | 1.5 | 1.0-2.0 | 1.4 | 0.9-1.9 | 1.5 | 1.0-2.1 | 2.7 | 1.0-4.4 | 1.2 | 0.9-1.5 | 1.3 | 0.9-1.7 | 0.9 | 0.4-1.3 | 0.9 | 0.6-1.1 |
| Tetracycline | 1.0 | 0.6-1.3 | 16.9 | 14.4-19.4 | 0.2 | 0.1-0.2 | 0.3 | 0.2-0.4 | 0.4 | 0.1-0.7 | 0.4 | 0.0-1.0 | 0.8 | 0.6-1.0 | 0.8 | 0.5-1.0 | 0.5 | 0.4-0.7 | 0.9 | 0.6-1.0 |

^a^ 95% Confidence interval.

^b^ Polymyxin b sulphate, mupirocin, novobiocin, thymol and bronopol.

^c^ Ampicillin and cloxacillin.

**Table 6**

Feline antimicrobial agent prescription (systemic and topical) by class as percentage of total prescriptions for each main presenting complaint in a network of United Kingdom small animal veterinary premises (data collected from 1 April 2014 to 31 March 2016).

| Antibiotic class | Gastroenteric | | Respiratory | | Pruritus | | Trauma | | Tumour | | Kidney disease | | Other unwell | | Post-operative | | Vaccination | | Other healthy | |
| --- | --- | --- | --- | --- | --- | --- | --- | --- | --- | --- | --- | --- | --- | --- | --- | --- | --- | --- | --- | --- |
|  | % | 95% CI ^a^ | % | 95% CI ^a^ | % | 95% CI ^a^ | % | 95% CI ^a^ | % | 95% CI ^a^ | % | 95% CI ^a^ | % | 95% CI ^a^ | % | 95% CI ^a^ | % | 95% CI ^a^ | % | 95% CI ^a^ |
| Aminoglycoside | 0.8 | 0.4-1.2 | 1.3 | 0.9-1.6 | 8.3 | 7.3-9.2 | 1.5 | 1.2-1.9 | 1.5 | 0.8-2.2 | 0.7 | 0.2-1.3 | 5.0 | 4.6-5.5 | 3.8 | 2.8-4.7 | 13.5 | 11.8-15.3 | 6.1 | 5.2-7.0 |
| Amphenicol | 0.1 | 0.0-0.2 | 1.0 | 0.6-1.3 | 0.3 | 0.1-0.5 | 0.7 | 0.5-0.8 | 0.3 | 0.0-0.6 | 0.3 | 0.0-0.6 | 2.0 | 1.7-2.3 | 1.0 | 0.7-1.4 | 2.0 | 1.4-2.5 | 1.6 | 1.1-2.0 |
| Other agent ^b^ | 1.0 | 0.0-2.1 | 0.7 | 0.4-1.0 | 5.7 | 4.8-6.6 | 0.6 | 0.5-0.7 | 1.1 | 0.5-1.7 | 0.7 | 0.1-1.3 | 3.0 | 2.7-3.3 | 2.3 | 1.7-2.9 | 7.0 | 5.8-8.2 | 3.8 | 3.2-4.3 |
| β-lactam (total) | 71.2 | 67.3-75.2 | 74.5 | 71.7-77.2 | 62.2 | 59.6-64.8 | 85.3 | 83.2-87.4 | 79.8 | 76.8-83.8 | 90.9 | 88.7-93.2 | 68.1 | 66.4-69.7 | 71.7 | 69.7-73.7 | 41.5 | 37.5-45.5 | 65.8 | 63.5-68.2 |
| Amoxicillin | 26.3 | 22.4-30.2 | 8.5 | 6.1-10.9 | 5.4 | 3.3-7.4 | 14.1 | 11.0-17.2 | 11.5 | 8.8-14.2 | 10.4 | 7.4-13.3 | 12.3 | 9.9-14.8 | 12.8 | 10.0-15.5 | 6.1 | 3.9-8.3 | 13.7 | 8.1-19.3 |
| Other β-lactam ^c^ | 0.1 | 0.0-0.2 | 0.1 | 0.0-0.2 | 0.1 | 0.0-0.2 | 0.1 | 0.0-0.2 | 0.0 | <0.00 | 0.0 | <0.00 | 0.08 | 0.00-0.17 | 0.03 | 0.00-0.08 | 0.1 | 0.0-0.3 | 0.05 | 0.0-0.09 |
| First generation cephalosporin | 0.03 | 0.00-0.08 | 0.3 | 0.1-0.5 | 1.5 | 1.0-2.0 | 0.4 | 0.2-0.5 | 0.5 | 0.1-0.9 | 0.4 | 0.0-1.1 | 0.3 | 0.2-0.4 | 0.3 | 0.1-0.5 | 0.3 | 0.1-0.6 | 0.3 | 0.2-0.5 |
| Second generation cephalosporin | 0.0 | <0.00 | 0.0 | <0.00 | 0.0 | <0.00 | 0.04 | 0.00-0.07 | 0.0 | <0.00 | 0.1 | 0.0-0.4 | 0.0 | <0.00 | 0.05 | 0.00-0.12 | 0.0 | <0.00 | 0.01 | 0.00-0.04 |
| Third generation cephalosporin | 17.5 | 14.9-20.1 | 41.8 | 38.5-45.1 | 46.0 | 43.2-48.9 | 39.9 | 35.9-43.9 | 49.2 | 45.0-53.4 | 56.8 | 51.7-61.9 | 35.0 | 32.5-37.5 | 34.2 | 30.9-37.4 | 23.5 | 20.3-26.6 | 34.4 | 30.3-38.4 |
| Clavulanic acid potentiated amoxicillin | 27.3 | 24.5-30.1 | 23.8 | 20.9-26.7 | 9.2 | 7.7-10.6 | 30.7 | 27.0-34.5 | 18.6 | 15.6-21.7 | 23.3 | 18.9-27.8 | 20.4 | 18.5-22.2 | 24.4 | 21.6-27.2 | 11.4 | 9.2-13.6 | 17.4 | 14.3-20.4 |
| Penicillin | 0.2 | 0.0-0.4 | 0.04 | 0.00-0.10 | 0.04 | 0.00-0.09 | 0.01 | 0.00-0.03 | 0.0 | <0.00 | 0.0 | <0.00 | 0.02 | 0.00-0.03 | 0.1 | 0.0-0.2 | 0.0 | <0.00 | 0.04 | 0.00-0.09 |
| Fluoroquinolone | 3.9 | 0.3-7.5 | 6.3 | 3.4-9.2 | 1.4 | 0.9-1.9 | 2.3 | 0.3-4.3 | 3.3 | 1.6-5.0 | 2.7 | 1.2-4.2 | 3.3 | 2.0-4.6 | 3.0 | 2.3-3.7 | 1.9 | 1.0-2.8 | 2.6 | 1.7-3.5 |
| Fusidic acid | 2.0 | 1.5-2.4 | 6.5 | 5.6-7.4 | 20.0 | 18.4-21.5 | 4.7 | 4.2-5.1 | 5.6 | 4.3-6.8 | 2.4 | 1.4-3.5 | 11.9 | 11.2-12.6 | 9.1 | 8.0-10.3 | 28.6 | 26.2-31.0 | 13.2 | 11.9-14.5 |
| Lincosamide | 1.4 | 0.5-2.3 | 1.1 | 0.7-1.5 | 1.9 | 1.3-2.6 | 4.7 | 3.7-5.6 | 7.0 | 4.8-9.2 | 1.9 | 1.0-2.9 | 4.8 | 4.0-5.6 | 7.2 | 5.8-8.6 | 3.3 | 2.5-4.1 | 4.3 | 3.5-5.1 |
| Macrolide | 0.5 | 0.0-1.0 | 0.2 | 0.0-0.3 | 0.0 | <0.00 | 0.0 | <0.00 | 0.0 | <0.00 | 0.0 | <0.00 | 0.02 | 0.00-0.05 | 0.03 | 0.00-0.07 | 0.06 | 0.00-0.13 | 0.05 | 0.00-0.10 |
| Nitroimidazole | 14.6 | 11.8-17.3 | 0.3 | 0.0-0.6 | 0.1 | 0.0-0.2 | 0.13 | 0.05-0.22 | 0.7 | 0.2-1.3 | 0.4 | 0.0-0.8 | 0.7 | 0.6-0.9 | 0.8 | 0.5-1.2 | 0.8 | 0.3-1.2 | 1.1 | 0.7-1.6 |
| Nitroimidazole-macrolide | 3.7 | 2.2-5.2 | 0.06 | 0.00-0.14 | 0.0 | <0.00 | 0.01 | 0.00-0.02 | 0.1 | 0.0-0.2 | 0.0 | <0.00 | 0.2 | 0.1-0.3 | 0.3 | 0.1-0.4 | 0.4 | 0.1-0.6 | 0.6 | 0.2-1.0 |
| Rifamycin | 0.03 | 0.00-0.08 | 0.0 | <0.00 | 0.0 | <0.00 | 0.0 | <0.00 | 0.0 | <0.00 | 0.0 | <0.00 | 0.0 | <0.00 | 0.0 | <0.00 | 0.0 | <0.00 | 0.0 | <0.00 |
| Sulphonamide | 0.2 | 0.1-0.4 | 0.09 | 0.00-0.19 | 0.1 | 0.0-0.2 | 0.01 | 0.00-0.02 | 0.0 | <0.00 | 0.0 | <0.00 | 0.05 | 0.02-0.09 | 0.03 | 0.00-0.08 | 0.0 | <0.00 | 0.01 | 0.00-0.03 |
| Tetracycline | 0.6 | 0.1-1.0 | 8.1 | 6.7-9.5 | 0.1 | 0.0-0.2 | 0.1 | 0.0-0.2 | 0.6 | 0.1-1.0 | 0.0 | <0.00 | 1.0 | 0.8-1.1 | 0.7 | 0.4-1.0 | 1.1 | 0.7-1.4 | 0.9 | 0.7-1.2 |

^a^ 95% Confidence interval.

^b^ Polymyxin b sulphate, mupirocin, novobiocin, thymol and bronopol.

^c^ Ampicillin and cloxacillin.

**Supplementary Fig. 1**

Geographical representation of UK small animal veterinary premise locations (red points) that have contributed data to the Small Animal Veterinary Surveillance Network (SAVSNET) project.

**
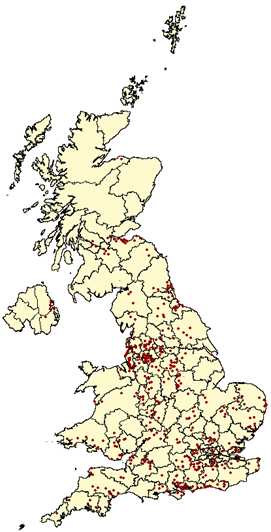
**
